# Hyperrealistic neural decoding for reconstructing faces from fMRI activations via the GAN latent space

**DOI:** 10.1038/s41598-021-03938-w

**Published:** 2022-01-07

**Authors:** Thirza Dado, Yağmur Güçlütürk, Luca Ambrogioni, Gabriëlle Ras, Sander Bosch, Marcel van Gerven, Umut Güçlü

**Affiliations:** Donders Institute for Brain, Cognition and Behaviour, Radboud University, Nijmegen, The Netherlands

**Keywords:** Computational neuroscience, Visual system

## Abstract

Neural decoding can be conceptualized as the problem of mapping brain responses back to sensory stimuli via a feature space. We introduce (i) a novel experimental paradigm that uses well-controlled yet highly naturalistic stimuli with a priori known feature representations and (ii) an implementation thereof for HYPerrealistic reconstruction of PERception (HYPER) of faces from brain recordings. To this end, we embrace the use of generative adversarial networks (GANs) at the earliest step of our neural decoding pipeline by acquiring fMRI data as participants perceive face images synthesized by the generator network of a GAN. We show that the latent vectors used for generation effectively capture the same defining stimulus properties as the fMRI measurements. As such, these latents (conditioned on the GAN) are used as the in-between feature representations underlying the perceived images that can be predicted in neural decoding for (re-)generation of the originally perceived stimuli, leading to the most accurate reconstructions of perception to date.

## Introduction

**Figure 1:**
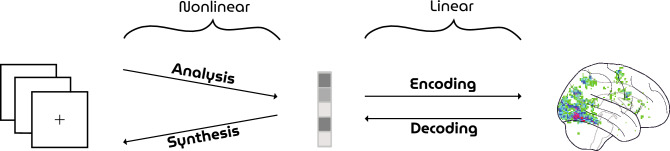
Neural coding. The mapping between sensory stimuli (left) and brain measurements (right) via a feature space (middle). Neural encoding seeks to find a transformation from stimulus to the observed brain response. Conversely, neural decoding seeks to find the information present in the observed brain responses by a mapping from brain activity back to the originally perceived stimulus.

Neural decoding can be conceptualized as the inverse problem of mapping brain responses back to sensory stimuli via a feature space^1^. Such a mapping can be modeled as a composite function of linear and nonlinear transformations (Fig. 1). A nonlinear transformation models the stimulus-feature mapping whereas the feature-response mapping is modeled by a linear transformation. Invoking this in-between feature space factorizes the direct stimulus-response transformation into two to make it not only data efficient (given that neural data is scarce) but also possible to test alternative hypotheses about the emergence and nature of neural representations of the environment. That is, each stimulus-feature model transforms stimuli into a different set of underlying features to construct candidate feature representations. Each feature-response model then linearly transforms these candidate feature representations into brain responses to test similarity thereof. Feature representations of stimuli are assumed to have a linear relationship with neuroimaging measurements of underlying neural responses in that both capture the same statistical invariances in the environment.

The systematic correspondence between various feature representations of discriminative task-optimized (supervised) deep neural networks and neural representations of sensory cortices are well-established^2–7^. As such, exploiting this correspondence in neural decoding of visual perception has pushed the state-of-the-art forward^1^ such as classification of perceived, imagined and dreamed object categories^8,9^, and reconstruction of perceived natural images^10,11^, movies^12^ and faces^13,14^. However, unlike their supervised counterparts, more biologically plausible unsupervised deep neural networks have paradoxically been less successful in modeling neural representations^15^.

At the same time, generative adversarial networks (GANs)^16^ have emerged as perhaps the most powerful generative models to date^17–19^ that can potentially bring neural decoding to the next level. In short, a generator network is pitted against a discriminator network that learns to distinguish synthesized from real data. The goal of the generator is to fool the discriminator by mapping “latent” vector samples from a given (simple) distribution (e.g., standard Gaussian) to unique data samples that appear to have been drawn from the real data distribution. This competition drives the networks to improve in tandem until the generator has learned the unidirectional mapping from latent to data distribution such that the synthesized samples are indistinguishable from the real ones. Importantly, this mapping can model the synthesis operation (i.e., the nonlinear feature-stimulus transformation as defined under neural decoding) where the latent vectors *are* the feature representations underlying the stimuli.

For this reason, GANs have high potential in modeling neural representations but testing this hypothesis is not directly possible because latents cannot be obtained retrospectively; arbitrary stimuli cannot be directly transformed into latents since GANs do not have such an inverse transformation due to the nonlinearities of the (unidirectional) network. Hence, unlike the aforementioned discriminative convnets which are feature extractors by definition, the adoption of GANs in neural decoding has been relatively slow since they cannot be readily used for this purpose without resorting to approximate inversion methods (see^10^ for such an earlier attempt). In that case, the feature-stimulus transformation entails information loss as the data need to be reconstructed from the predicted feature representations using an approximate inversion network, leading to a severe bottleneck to the maximum possible reconstruction quality (i.e., the noise ceiling).

**Figure 2:**
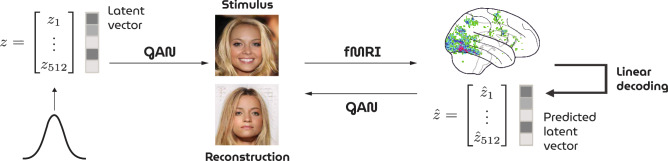
Illustration of the HYPER pipeline. Face images are generated from randomly sampled latent vectors *z* by a GAN and presented as stimuli during brain scanning. A linear model predicts latent vectors $\hat{z}$ for unseen brain responses to feed back to the GAN for reconstruction.

We overcome the aforementioned problem by introducing a very powerful yet simple experimental paradigm for neural decoding where participants are presented with synthetic yet highly naturalistic stimuli with known latent vectors. We also present a model instance of this paradigm for HYperrealistic reconstruction of PERception (HYPER) which elegantly integrates GANs in neural decoding of faces by combining the following components (Fig. 2):

- A pretrained generator of a progressive growing of GAN (PGGAN)^17^ that generates photorealistic faces from latents.
- A new dataset of synthesized face images and whole-brain fMRI activations of two participants.
- A decoding model that predicts latents from fMRI activations which are fed to the generator for synthesis/reconstruction.

We demonstrate that our approach constitutes a leap forward in our ability to reconstruct percepts from patterns of human brain activity.

**Figure 3:**
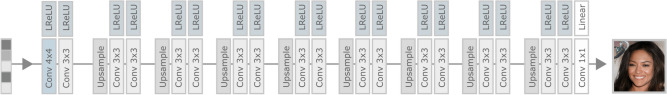
PGGAN generator network. The architecture consists of nine blocks with a total of 23.1 M trainable parameters. It transforms 512-dimensional Gaussian latent vectors into high-resolution RGB face images ($1024 \times 1024$ pixels).

## Methods

### HYPER pipeline

An illustration of the HYPER pipeline can be found in Fig. 2. Visual face stimuli were synthesized by the generator network of a GAN and presented to participants in an fMRI scanner. Neural decoding was performed as follows: the generator network of the GAN was extended with a dense layer at the beginning of the network that performed the response-feature transformation (i.e., from voxel recordings to latent vectors). This response-feature layer was trained by iteratively minimizing the Euclidean distance between ground-truth and predicted latent vectors with the Adam optimizer until convergence while keeping the rest of the network fixed (batch size $= 30$, learning rate $= 0.00001$, weight decay $= 0.01$). Finally, the generator output were the reconstructed faces from brain activity.

### Datasets

#### Visual stimuli

High-resolution face images ($1024 \times 1024$ pixels) are synthesized by the pretrained generator network (Fig. 3) of a Progressive GAN (PGGAN) model^17^ from 512-dimensional latent vectors that are randomly sampled from the standard Gaussian. Each generated face image is cropped and resized to $224 \times 224$ pixels. Note that none of the face images in this manuscript are of real people. They are instead synthesized by a generative model that is trained on the large-scale face dataset *CelebFaces Attributes Dataset* (CelebA) that consists of more than 200 K celebrity images^20^.

#### Brain responses

**Figure 4:**
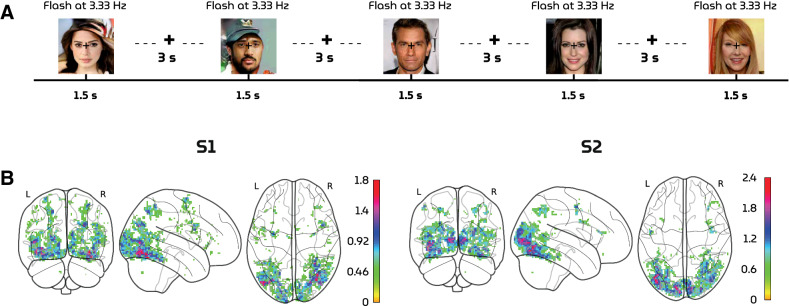
(**A**) Experimental paradigm. Visual stimuli were flashed with a frequency of 3.33 Hz for 1.5 s followed by an interstimulus interval of 3 s. (**B**) Voxel masks. The 4096 most active voxels were selected based on the highest z-statistics within the averaged z-map from the training set responses.

fMRI data were collected from two healthy participants (S1: 30-year old male; S2: 32-year old male) while they were fixating a center target ($0.6 \times 0.6$ degrees visual angle)^21^ superimposed on the face stimuli ($15 \times 15$ degrees visual angle) to minimize involuntary eye movements. The fMRI recordings were acquired with a multiband-4 protocol (TR $= 1.5$ s, voxel size $= 2 \times 2 \times 2$ mm$^3$, whole-brain coverage) in nine runs. Per run, 175 faces were presented that were flickering with a frequency of 3.33 Hz for 1.5 s, followed by an inter-stimulus interval of 3 s (Fig. 4A). The test and training set stimuli were presented in the first three and the remaining six runs, respectively. In total, 36 faces were repeated $\sim 14$ times for the test set and 1050 unique faces were presented once for the training set. This ensured that the training set covers a large stimulus space to fit a general face model whereas the voxel responses from the test set contain less noise and higher statistical power.

During preprocessing, the brain volumes were realigned to the first functional scan and the mean functional scan, respectively, after which the volumes were normalized to MNI space. A general linear model was fit to deconvolve task-related neural activation with the canonical hemodynamic response function. Next, we computed the t-statistic for each voxel which was standardized to obtain brain maps in terms of z-scores. The most active 4096 voxels on average were selected from the training set to define a voxel mask (i.e., voxels were selected based on amplitude rather than significance) (Fig. 4B). Voxel responses from the test set were not used to create this mask to avoid circularity. To inspect contributions of different brain areas to linear decoding, we included the voxel distribution across the 22 main cortical brain regions according to the HCP MMP 1.0 atlas^22^ in the supplementary materials. Among the voxels that are part of the atlas, most contributions were from those in the ventral stream followed by MT+ and vicinity and early visual cortex.

The experiment was approved by the local ethics committee (CMO Regio Arnhem-Nijmegen). Subjects provided written informed consent in accordance with the Declaration of Helsinki.

### Evaluation

Model performance was evaluated in terms of three metrics: latent similarity, feature similarity and Pearson correlation. First, latent similarity is the Euclidean similarity between predicted and true latent vectors. Concretely, let $\hat{z}$ and *z* be the 512-dimensional predicted and true latent vectors, respectively. Latent similarity is then defined as follows:

$$\begin{aligned} {\text {Latent\, Similarity}} = \frac{1}{\sqrt{\sum _{i = 1}^{512} \left( \hat{z}_i - z_i\right) ^2} + 1} \end{aligned}$$

Second, feature similarity is the Euclidean similarity between feature extraction layer outputs ($n=2048$) of the ResNet50 model, pretrained for face recognition. Concretely, let *x* and $\hat{x}$ be the $224 \times 224$ RGB images of stimuli and their reconstructions, respectively. Furthermore, let *f*(.) be the 2048-dimensional features of the ResNet50 model pretrained on face recognition. Feature similarity is then defined as follows:

$$\begin{aligned} {\text {Feature\, Similarity}} = \frac{1}{\sqrt{\sum _{i = 1}^{2048} \left( f(\hat{x})_i - f(x)_i\right) ^2} + 1} \end{aligned}$$

Third, Pearson correlation measures the standard linear (product-moment) correlation between the luminance pixel values of stimuli and their reconstructions.

**Figure 5:**
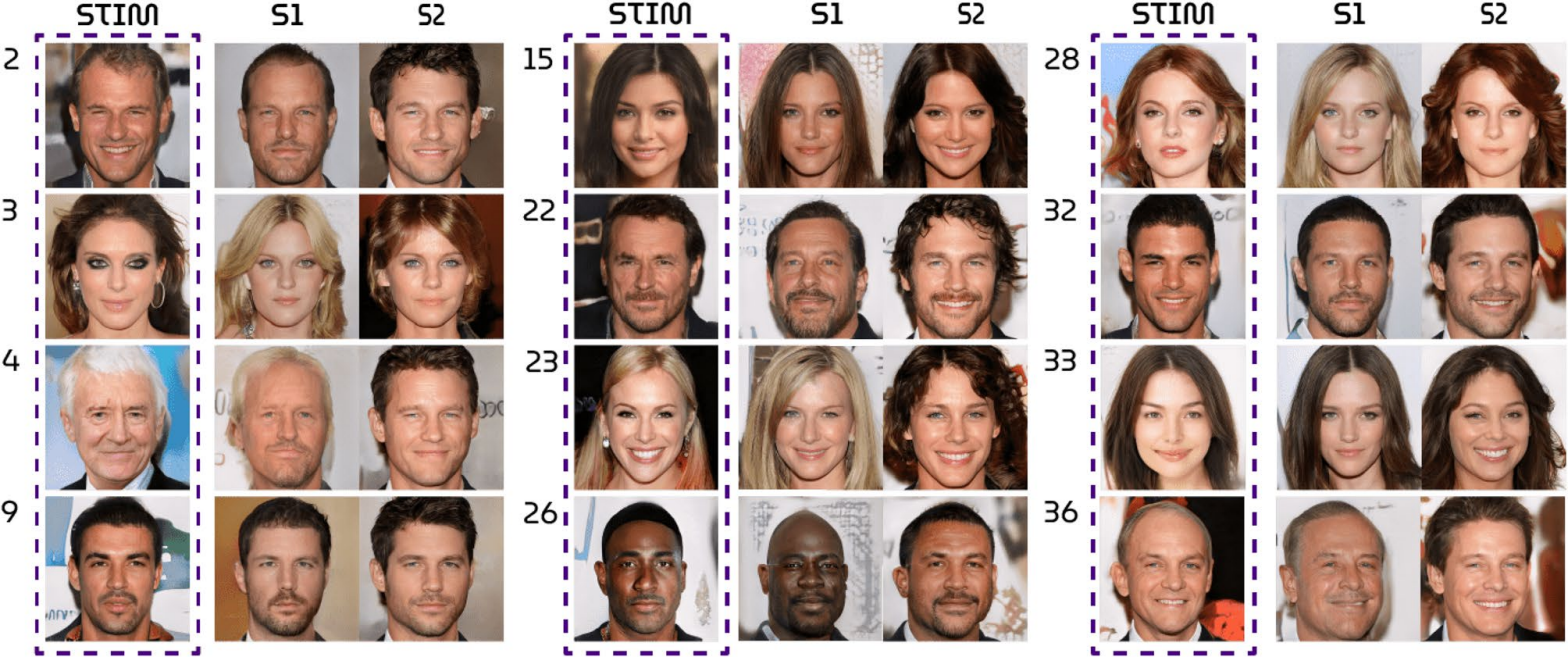
Stimulus-reconstructions. The three blocks show twelve arbitrarily chosen but representative test set examples. The first column displays the face stimuli whereas the second and third column display the corresponding reconstructions from brain activations from subject 1 and 2, respectively.

Additionally, we also introduce a novel metric which we call attribute similarity. Based on the assumption that there exists a hyperplane in latent space for binary semantic attributes (e.g., male vs. female), Shen et al.^23^ have identified the decision boundaries for semantic face attributes in PGGAN’s latent space by training independent linear support vector machines on gender, age, the presence of eyeglasses, smile, and pose. Attribute scores are then computed by taking the inner product between latents and decision boundary. In this way, model performance can be evaluated in terms of these specific visual attributes along a continuous spectrum.

### Implementation details

fMRI preprocessing is implemented in SPM12 after which first-order analysis is carried out in Nipy. We used a custom implementation of PGGAN in MXNet together with the pretrained weights from the original paper. A Keras pretrained implementation of VGGFace (ResNet50 model) is used to evaluate similarities between feature maps of the perceived and reconstructed images. The fMRI dataset for both subjects and used models are openly accessible (see supplementary materials).

### Ethical concerns

Care must be taken as “mind-reading” technologies also involve serious ethical concerns regarding mental privacy. Although current neural decoding approaches such as the one presented in this manuscript would not allow for involuntary access to thoughts of a person, future developments may allow for the extraction of information from the brain more easily, as the field is rapidly developing. As with all scientific and technological developments, ethical principles and guidelines as well as data protection regulations should be followed strictly to ensure the safety of potential users of these technologies.

## Results

Neural decoding of fMRI measurements via the GAN latent space has resulted in unprecedented reconstructions of perception. Figure 5 shows arbitrarily chosen but representative examples of stimuli and their reconstructions. The complete set of stimuli and reconstructions can be found in the supplementary materials.

**Table 1:** Quantitative results. Model performance of the HYPER model compared to the state-of-the-art VAE-GAN approach^14^ and the eigenface approach^24^ in terms of the feature similarity (column 2) and pearson correlation (column 3) between stimuli and reconstructions (mean ± std error). The first column displays latent similarity between true and predicted latents which is only applicable to the HYPER model. For a fair comparison, all images are resized to 224 $\times$ 224 pixels and backgrounds are removed. The statistical significance of HYPER is evaluated against randomly generated latent vectors and their synthesized images.

		Lat. sim.	Feat. sim.	Pearson. corr.
S1	HYPER	0.4722 ± 0.0024	0.1656 ± 0.0050	0.5464 ± 0.0256
		($p<0.001; perm. test$)	($p<0.001; perm. test$)	($p<0.001; perm. test$)
	VAE-GAN	–	0.1416 ± 0.0025	0.3354 ± 0.0400
	Eigenface	–	0.1319 ± 0.0016	0.4540 ± 0.0328
S2	HYPER	0.4666 ± 0.0020	0.1665 ± 0.0058	0.5013 ± 0.0291
		($p<0.001; perm. test$)	($p<0.001; perm. test$)	($p<0.001; perm. test$)
	VAE-GAN	–	0.1461 ± 0.0022	0.4137 ± 0.0353
	Eigenface	–	0.1261 ± 0.0019	0.4267± 0.0297

**Figure 6:**
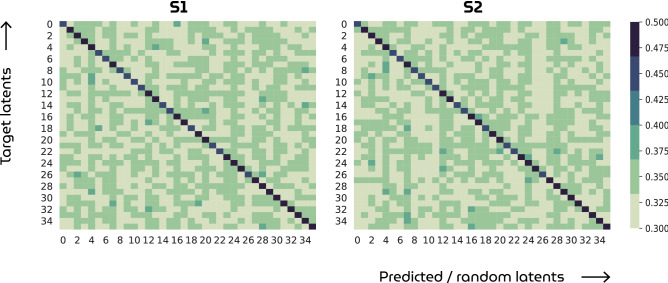
Latent similarity maps. The diagonal displays the similarity between target and predicted latent vectors whereas off-diagonal entries display similarity between targets and randomly sampled latents from the same standard Gaussian distribution. The dark blue diagonal denotes that predictions always outperform random latents in terms of latent similarity.

**Figure 7:**
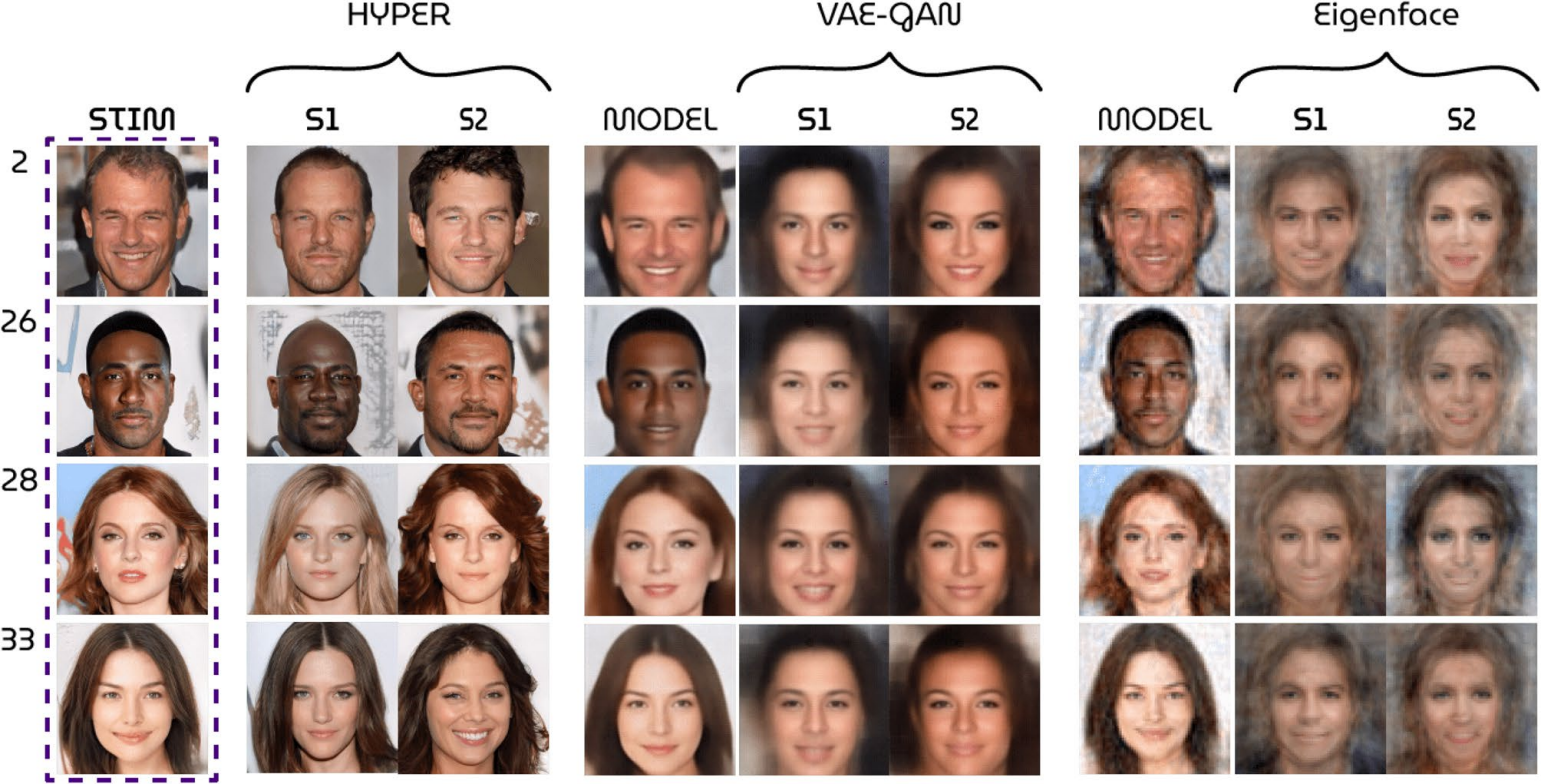
Qualitative results. Model performance of the HYPER model compared to VAE-GAN approach^14^ and the eigenface approach^24^. The *model* columns display the best possible results by direct encoding and decoding of the stimuli (i.e., noise ceiling; no brain data is used for these reconstructions). For HYPER, the stimuli themselves are the best possible results.

The performance of the HYPER model was compared to two baseline models that map the brain recordings onto different latent spaces. The first baseline was the state-of-the-art variational autoencoder of 13 layers which was trained adversarially with a discriminator network (VAE-GAN)^14^ to reconstruct CelebA faces ($128 \times 128$ pixels) from 1024-dimensional latents. Note that the pretrained network from the original paper was used. Moreover, representational similarity analysis between the PGGAN and VAE-GAN latent spaces revealed these to be significantly dissimilar ($r = 0.0063$, $p \ll 0.05$, Student’s t-test). The second baseline was the traditional eigenface approach^24^ that predicted the first 512 principal components (or “eigenfaces”) and reconstructed face images ($64 \times 64$ pixels) by applying an inverse PCA transform. For a fair comparison, the same voxel masks were used to evaluate all three methods presented in this study without any optimization to a particular decoding approach.

All quantitative (Table 1) and qualitative (Fig. 7) results showed that the HYPER model outperformed the baselines. Furthermore, a permutation test was performed where we randomly generated 1000 latent vectors from the same distribution as the target vectors and compared how similar the targets were to the predictions versus to the randomly generated latents. The targets were more similar to the predictions than to the randomly generated latents with $p < 0.001$. Figure 6 shows similarity maps of this analysis with 35 randomly generated latents per target instead of 1000 for visualization purposes. A similar permutation test was also performed for the other feature similarity and correlation metrics with the same significance results.

**Figure 8:**
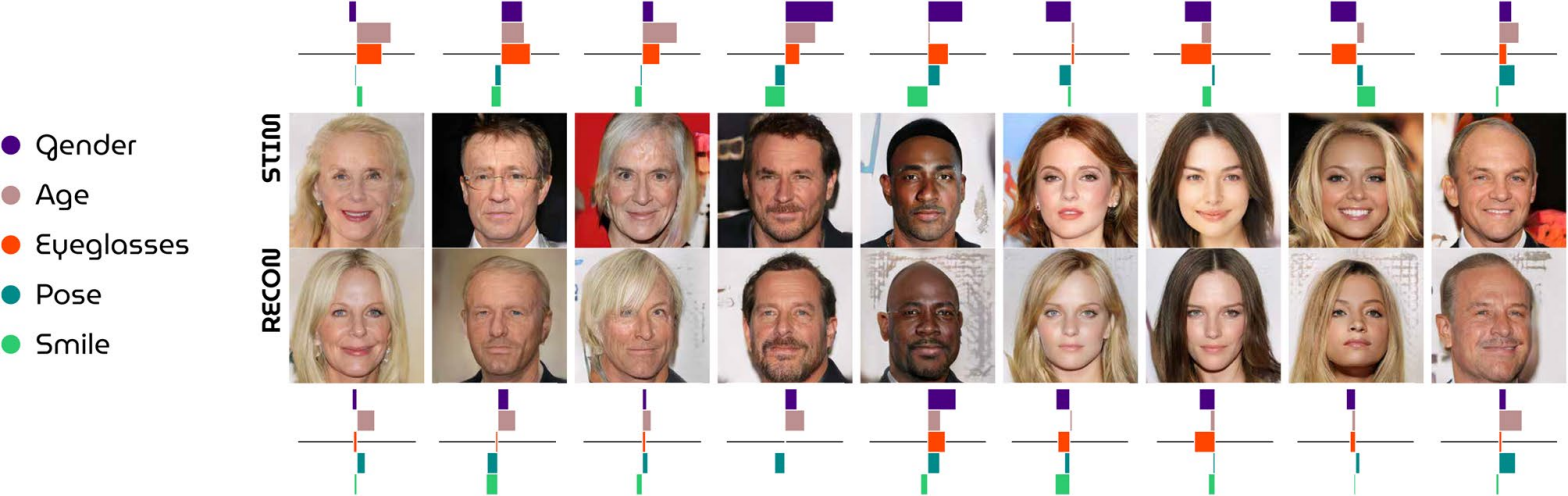
Attribute scores. Stimulus-reconstruction examples (subject 1) with rotated bar graphs denoting the attribute scores for gender, age, eyeglasses, pose and smile to visually demonstrate how this metric can be used to evaluate model performance with respect to semantic face attributes.

**Figure 9:**
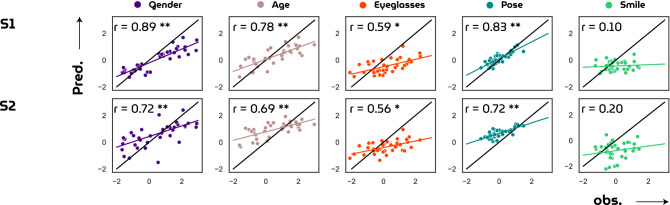
Attribute reconstruction performance. The correlation coefficients between observed and predicted target scores are found to be highly significant for gender, age and pose ($p\ll 0.05$; Student’s t-test), significant for eyeglasses ($p < 0.05$; Student’s t-test) and not significant for smile ($p>> 0.05$; Student’s t-test).

Next, Fig. 8 illustrates how well HYPER decoded face attributes by matching polarity and intensity of attribute scores between perceived and reconstructed examples. For most stimulus-reconstruction pairs, the graphs match in terms of directionality. Correlating observed and predicted attribute scores resulted in significant ($p \ll 0.05$; Student’s t-test) results for gender, age, eyeglasses and pose, but not for smile (Fig. 9).

**Figure 10:**
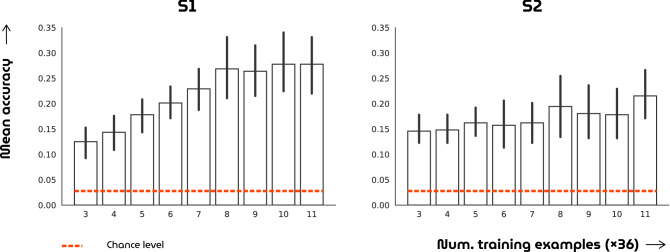
Reliability of brain recordings. The bar graphs show the mean classification accuracy with standard deviation (Y axis) for nine classifiers (X axis) that are trained on an increasing number of brain volume repetitions. The dotted line denotes chance level.

Lastly, the reliability of the fMRI recordings was addressed using twelve single repetitions per face image from the test set (Fig. 10). Here, L2-regularized multiclass logistic regression with nested 3- and 12-fold cross-validation was carried out to classify face images from brain volumes. The inner three folds were used for estimating the regularization coefficient whereas the outer 12 folds were used for estimating the reliability. For each of the outer 12 folds, nine separate classifiers were trained on an increasing number of repetitions ranging from three to eleven and tested on single repetitions. As expected, the highest accuracy was achieved when the maximum number of repetitions were used. At the same time, all classifiers performed significantly above chance-level regardless of the number of repetitions (p< 0.05, Student’s t-test).

## Discussion

The novel experimental paradigm for neural decoding that we introduced uses synthesized yet hyperrealistic stimuli such that the underlying latent/feature representations needed for (re)generation are known a priori. The HYPER model is an implementation of this paradigm which has decoded fMRI recordings into the best reconstructions of perceived face images to date using the generator of a GAN that is capable of synthesizing photorealistic faces from latent vectors. The results indicate that unsupervised deep neural networks can successfully model neural representations of naturalistic stimuli and that the GAN latent space approximates the neural face manifold. We would like to note however that this does not mean a relationship between arbitrary Gaussian variables and brain activity rather one between Gaussian latents conditioned on the GAN and brain activity.

HYPER achieved considerably better reconstructions than the two baselines. Importantly, not only do we attribute the high performance of HYPER to the type of generative model but especially to the training on synthesized yet photorealistic faces; having access to the ground-truth latent vectors from the get-go was crucial in exploiting the benefits of GANs in neural decoding rather than relying on approximate inference to obtain them as VAE-GANs do by design and GANs can do by post hoc modification^10^. It should also be noted that the reconstructions by the VAE-GAN approach appear to be of lower quality than those presented in the original study. Likely explanations for this are the differences in dataset size and the voxel selection procedure.

### Limitations of HYPER

While HYPER owes its performance to the current advances in generative modeling, it also inherits the limitations thereof. So far, HYPER has been evaluated by reconstructing synthetic faces from fMRI measurements. The next step is verifying whether a decoding model trained on brain responses during synthetic face perception generalizes to faces of real people. Latent vectors of real faces are not directly accessible but would also no longer be required when the decoding model has learned to accurately predict them from the synthetic data. It should however be noted that the results of this study are already valid reconstructions of visual perception regardless of the nature of the stimuli themselves.

Reconstructions by HYPER appear to contain biases. First, the linear model predicts primarily latent vectors corresponding to young, western-looking faces without eyeglasses as they tend to follow the image statistics of the (celebrity) training set. Second, the PGGAN generator is known to suffer from the problem of *feature entanglement* where manipulating one particular feature in latent space affects other features as well^23^. For instance, editing a latent vector to make the generated face wear eyeglasses simultaneously tends to make the face look older because of such biases in the training data. Feature entanglement obstructs the generator to map unfamiliar latent elements to their respective visual features. It is easy to foresee potential complications for reconstructing images of real faces.

A modified version of PGGAN, called StyleGAN^19,25^, is designed to overcome the feature entanglement problem. StyleGAN maps the entangled latent vector to an additional intermediate latent space (thereby reducing feature entanglement) which is then integrated into the generator network using adaptive instance normalization. This results in superior control over the features in the reconstructed images and possibly the generator’s ability to reconstruct unfamiliar features. The generated face photographs by StyleGAN have improved considerably in quality and variation in comparison to PGGAN. Replacing PGGAN with StyleGAN would therefore be a logical next step for studies concerned with the neural decoding of faces.

Finally, this study used a dataset with many trials but from a small number of participants as was the case in earlier similar studies^8,10–14,26,27^. Our goal was to investigate how well GANs can be used to reconstruct perceived stimuli from fMRI measurements of individual participants. As such, all analyses were performed separately for individual participants. Our results demonstrated that our framework can indeed be successfully used to create hyperrealistic reconstructions of perceived faces for these participants. However, it should be noted that a larger group study would be required to generalize our conclusions to the whole population.

### Future applications

The field of neural decoding has been gaining more and more traction in recent years as advanced computational methods became increasingly available for application on neural data. This is a very welcome development in both neuroscience and neurotechnology since reading neural information will not only help understand and explain human brain function but also find applications in brain computer interfaces and neuroprosthetics to help people with disabilities. For example, extensions of this framework to imagery could make it a preferred means for communication with locked-in patients.

## Conclusion

We have presented a novel experimental framework together with a model for HYperrealistic reconstruction of PERception (HYPER) by neural decoding of brain responses via the GAN latent space, leading to unparalleled stimulus reconstructions. Considering the speed of progress in the field of generative modeling, we believe that this framework will likely result in even more impressive reconstructions of perception and possibly even imagery in the near future.

## Supplementary Information

Supplementary Information.
