# Supplementary material for: Hyperrealistic neural decoding for reconstructing faces from fMRI activations via the GAN latent space
Source: Sci Rep. 2022 Jan 7;12:141. doi: 10.1038/s41598-021-03938-w (PMC8741893; doi:10.1038/s41598-021-03938-w)
Supplement: Supplementary file 1 — Supplementary Information. [file 41598_2021_3938_MOESM1_ESM.pdf]

Supplementary materials

A Github repository

https://github.com/neuralcodinglab/hyper

B Distribution of mask voxels across brain regions.

Table 1. Distribution of mask voxels across the 22 main cortical brain regions according to the HCP MMP 1.0 atlas[?]. The relative number of mask voxels in each brain region and their relative absolute weight amplitudes are displayed to measure their contribution to linear neural decoding. It can be noted that main contributions were from the ventral stream followed by MT+ and vicinity and early visual cortex.

	Subject 1		Subject 2	
	num. (%)	*weight (%)*	*num. (%)*	*weight (%)*
Primary visual cortex	3.96	3.97	4.79	4.91
Early visual cortex	6.30	6.39	8.23	8.43
Dorsal stream visual pathway	2.20	2.34	1.78	1.76
Ventral stream visual pathway	11.96	12.28	11.16	11.06
MT+ complex and neighboring visual areas	7.40	7.59	6.86	6.76
Somatosensory and motor cortices	0.15	0.13	0.00	0.00
Paracentral lobular and mid cingulate cortex	0.59	0.57	0.02	0.02
Premotor cortex	1.66	1.72	0.34	0.35
Posterior opercular region	0.12	0.13	0.00	0.00
Early auditory cortex	0.00	0.00	0.00	0.00
Auditory association cortex	0.00	0.00	0.05	0.04
Insular and frontal opercular cortices	0.46	0.46	0.00	0.00
Medial temporal cortex	0.05	0.05	0.22	0.25
Lateral temporal cortex	1.03	1.01	1.22	1.18
Temporo-parieto occipital junction	1.93	2.01	2.03	2.03
Superior parietal lobe	2.03	2.07	1.10	1.14
Inferior parietal lobe	0.42	0.42	1.46	1.43
Posterior cingulate cortex	0.00	0.00	0.07	0.08
Anterior cingulate and medial prefrontal cortices	0.02	0.02	0.05	0.05
Orbital and polar frontal cortices	0.10	0.10	0.02	0.05
Inferior frontal gyrus	0.39	0.38	0.63	0.62
Dorsolateral prefrontal cortex	0.22	0.24	0.51	0.55
Other	59.03	58.11	59.45	59.29

C Stimulus-reconstructions

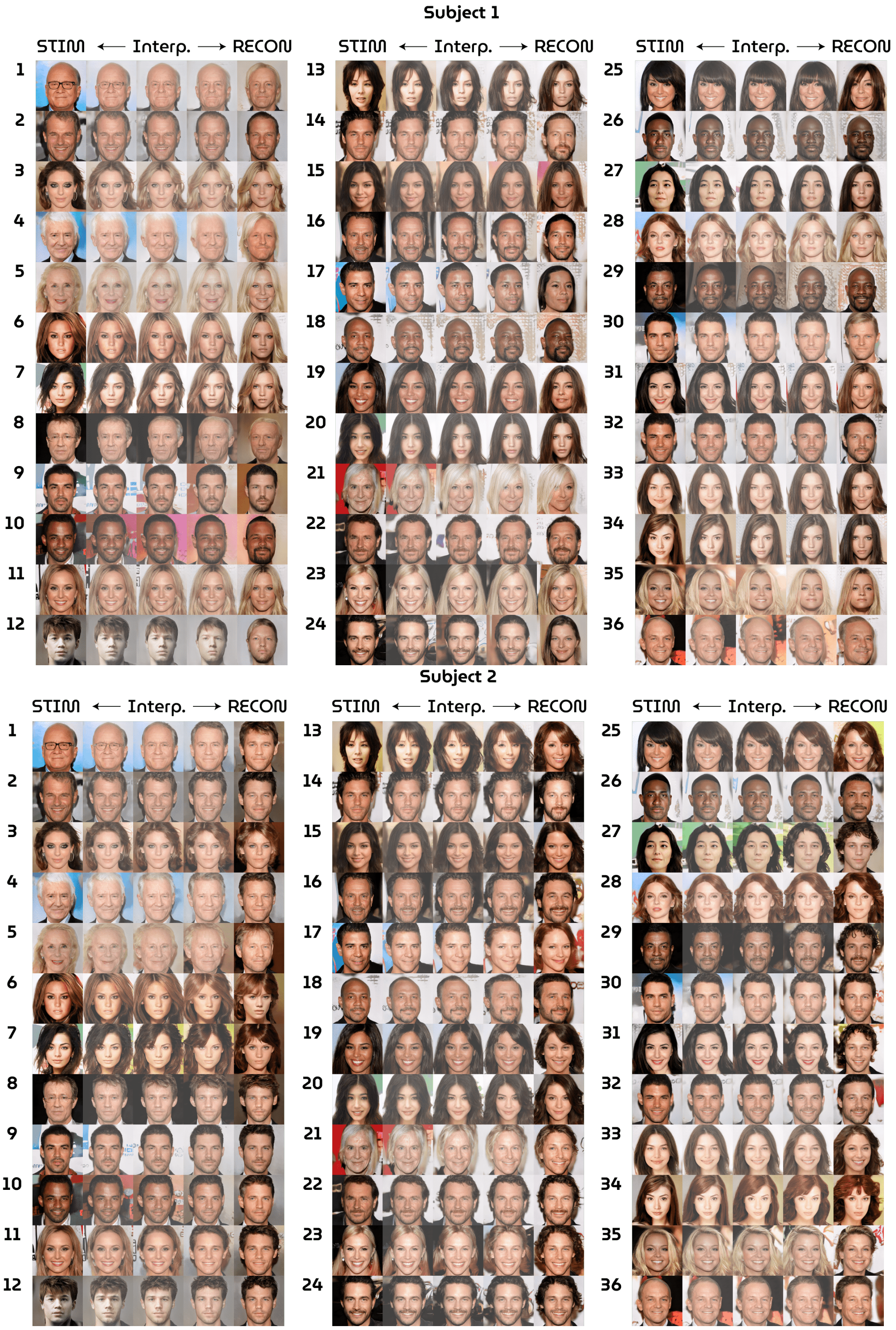

Figure 1. Stimuli (left) and reconstructions (right) for subject 1 and 2. The linear interpolations visualize the distance between predicted and true latent vector that underlie the (re)generated faces. In this way, it is easy to see which features are being retained or change.
